# Dense Calcification of the Common Femoral Artery Is Protective Against In-Stent Restenosis

**DOI:** 10.3390/jcm14197052

**Published:** 2025-10-06

**Authors:** Camil-Cassien Bamdé, Yann Goueffic, Jean-Michel Davaine, Alain Lalande, Charles Guenancia, Eric Steinmetz

**Affiliations:** 1 Cardiovascular and Thoracic Surgery Department, Dijon University Hospital, 21000 Dijon, France; eric.steinmetz@chu-dijon.fr; 2 Pathophysiology and Epidemiology of Cerebro-Cardiovascular Diseases (PEC2), EA 7460, Faculty of Health Sciences, University of Burgundy, 21000 Dijon, France; charles.guenancia@chu-dijon.fr; 3 Vascular and Endovascular Surgery Department, Paris Saint-Joseph Hospital, 75014 Paris, France; ygoueffic@ghpsj.fr; 4 Department of Vascular Surgery, Faculté de Médecine, Sorbonne Université, Assistance Publique-Hôpitaux de Paris (APHP), Hôpital Européen Georges Pompidou (HEGP), 20 rue Leblanc, 75015 Paris, France; jeanmichel.davaine@aphp.fr; 5 Department of Medical Imaging, Dijon University Hospital, 21000 Dijon, France; alain.lalande2@chu-dijon.fr; 6 ICMUB Laboratory, University of Burgundy, 21000 Dijon, France; 7 Cardiology Department, Dijon University Hospital, 21000 Dijon, France

**Keywords:** common femoral artery, femoral artery bifurcation, stenting, calcifications, restenosis

## Abstract

**Background:** Vascular calcification has been highlighted as a prognostic factor for perioperative thrombosis but a protective factor for late restenosis in lower limb peripheral artery disease (LLPAD). The aim of this study was to investigate the association between calcification and twelve-month primary patency in patients with stenting of the common femoral artery (CFA) and its bifurcation for atheromatous stenosis. **Materials/Methods:** This single-center retrospective study analyzed consecutive limbs (n = 90) that underwent CFA stenting for symptomatic lesions between January 2018 and January 2023. Calcification was assessed using dedicated computed tomography angiography analysis software (EndoSize; Therenva), with blinded evaluation of volume (mm^3^) and density (Hounsfield Units) across three anatomically distinct zones: proximal CFA (Zone 1); distal CFA (Zone 2); and bifurcation segments (Zone 3). The primary endpoint was twelve-month primary patency, defined as a peak systolic velocity ratio (PSVR) < 2.4 on duplex ultrasound without target lesion revascularization. Secondary endpoints included predictors of restenosis using multivariable logistic regression. **Results:** Ninety cases of CFA stenting for LLPAD (lower limb peripheral artery disease) were analyzed. A total of 78.9% of CFA lesions were treated for claudication and 21.1% for critical limb-threatening ischemia (CLTI). Lesions were distributed as Azema types I (1%), II (43%), and III (56%). At twelve-month follow-up, primary patency (PSVR < 2.4) was achieved in 77.4% of limbs. Patent CFA stenting demonstrated significantly higher median calcification density in Zone 2 compared to those with restenosis (1122 [IQR: 903–1248] vs. 858 [788–987] HU; *p* = 0.006; q = 0.021 after false discovery rate correction). ROC curve analysis identified a density threshold of 800 HU with a 76% reduction in restenosis risk (OR 0.24; 95% CI: 0.08–0.72; *p* = 0.011). Bootstrap validation (1000 replications) confirmed threshold stability at 821 HU (95% CI: 656–990 HU). **Conclusions:** In this exploratory study, dense calcification (≥800 HU) in the distal CFA appears to be protective against twelve-month restenosis following stenting. These findings suggest that calcification density may serve as a valuable predictor for patient selection and procedural planning in CFA interventions.

## 1. What This Paper Adds

Over the past decade, endovascular treatment of atheromatous lesions of the common femoral artery (CFA) and its bifurcation has evolved significantly to become an alternative to open surgery. The safety and efficacy of stenting the CFA and its bifurcation have been demonstrated in a randomized trial and various registries. However, patency appears to be influenced by parameters such as external compression (hip joint, sartorius) and arterial calcifications, which can be extensive. Here, we observe that arterial calcification is associated with a lower risk of restenosis after CFA stenting.

## 2. Introduction

Over the past decade, endovascular treatment of atheromatous lesions of the common femoral artery (CFA) and its bifurcation has evolved significantly, becoming a viable alternative to open surgery [1,2]. Recent multicenter registries have demonstrated good mid-term results [3] with substantially lower perioperative morbidity than traditional endarterectomy [4,5,6]. However, the durability of endovascular CFA interventions remains a major issue, with one-year primary patency rates [7] substantially lower than the long-term results achieved with surgical endarterectomy [8]. Highly calcified lesions are considered to be the most challenging to treat with endovascular techniques, leading to the development of dedicated tools, such as laser [9], orbital or rotational atherectomy [10], and even intravascular lithotripsy [11]. The exact clinical impact of calcification is still debated and varies greatly depending on the arterial location considered. As first described in interventional cardiology, severe calcification can lead to stent underexpansion [12] and compromise patency. Lee et al. [13] showed that it is a strong predictor of endovascular treatment failure. Kaladji et al. [14] found that severely calcified lesions had a higher risk of thrombosis but a lower rate of restenosis. The mechanisms underlying this protective calcification effect involve complex pathophysiological processes [15], whereby calcification characteristics (morphology, anatomical location (medial versus intimal), and volume) may influence treatment outcomes [14]. Histological studies have specifically documented the prevalence of fibrous lesions within CFA atherosclerotic plaques, frequently characterized by mature osteoid tissue formation that distinguishes them from the calcification patterns observed in other vascular territories [16,17]. Therefore, there is a need for more detailed analysis of calcifications and their clinical impact following endovascular treatment. While previous studies have established the general relationship between calcification and endovascular outcomes in various arterial territories [14], no prior investigation has quantitatively assessed the association between anatomically-specific calcification density (measured in Hounsfield Units, HU) and outcomes following CFA stenting. The existing literature has primarily relied on subjective angiographic scoring systems or global calcification burden assessments. Our study represents the first zone-specific quantitative analysis of calcification density in the CFA bifurcation, introducing a novel three-zone anatomical framework that accounts for the distinct biomechanical properties of each segment. Zone 2 (distal CFA) represents a biomechanically critical segment with reduced mobility during hip movement compared to Zone 1 (proximal CFA) and Zone 3 (ostias of superficial femoral artery SFA and deep femoral artery DFA). This zone-specific approach addresses a fundamental gap in current knowledge by providing objective, reproducible measurements that can guide clinical decision-making and risk stratification in CFA interventions.

## 3. Methods

### 3.1. Study Design

This was a single-center retrospective study conducted in a tertiary vascular surgery center. The data was obtained by querying the electronic medical records with the keywords: “endovascular”, “common femoral artery”, “deep femoral artery”, “stenting”, and “femoral bifurcation”. Patients were included if they presented with symptomatic atheromatous lesions of the CFA and its bifurcation treated with stenting between January 2018 and January 2023. Patients lost to follow-up were excluded from the final analysis. Ethical approval was not sought, in line with institutional policy on retrospective studies, and our work was completed in accordance with the Declaration of Helsinki as revised in 2013. All patients were informed of and consented to the anonymous processing of their data. Approval of the institutional committee was obtained, and this study was registered on HealthDataHub N°20230321114617, approved on 3 March 2023.

### 3.2. Patients

Patients with stenting of the CFA and its bifurcation for symptomatic atheromatous stenosis (Rutherford [18] 1 to 6) were included in the study. Stenting of the CFA and its bifurcation was defined as the placement of any stent in the CFA with extension to the deep femoral artery (DFA) or to the origin of the superficial femoral artery (SFA). Lesions of the CFA and its bifurcation were graded according to the Azema Classification [7]. Briefly, type I lesions are located at the iliofemoral junction and do not extend to the distal CFA and femoral bifurcation. Type II lesions are in the CFA trunk without proximal extension or involvement of the femoral bifurcation. Type III lesions are the most extensive, involving the CFA bifurcation. Finally, type IV lesions include proximal or distal anastomotic stenosis of bypass grafts. Patients were excluded if they had: a type IV lesion, prior open CFA surgery, percutaneous atherectomy of the CFA and its bifurcation, stenting for pseudoaneurysm, or stenting for non-atheromatous lesions (radiation-induced stenosis, closure system-related stenosis). Patients were also excluded if they had previous iliofemoral junction stenting, which could interfere with evaluation of the index procedure, defined here as the first endovascular procedure aiming to treat native CFA lesions. Vessel preparation consisted of balloon inflation with a diameter one millimeter below the nominal vessel diameter for two minutes. Balloon-expandable stents were preferred to manage the diameter mismatch between CFA and DFA/SFA, especially for post-dilation of the proximal part, to fit the nominal diameter of the CFA, and to ensure their precise deployment.

### 3.3. Quantification of Calcifications

**Figure 1:**
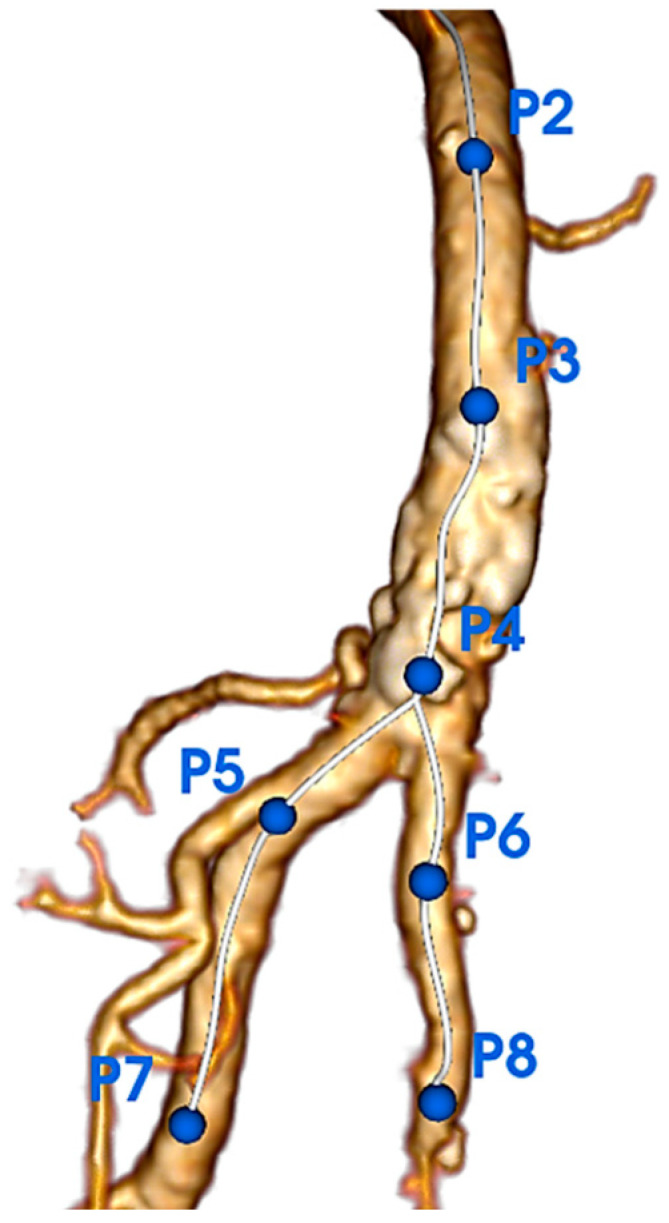
Segmentation of the common femoral artery (CFA) and its bifurcation showing anatomical landmarks. P2 = epigastric artery origin; P3 = midpoint between epigastric artery and femoral bifurcation; P4 = CFA bifurcation; P5 = 2 cm distal to deep femoral artery (DFA) ostium; P6 = 2 cm distal to superficial femoral artery (SFA) ostium. This standardized approach ensures reproducible zone definitions across all patients.

**Figure 2:**
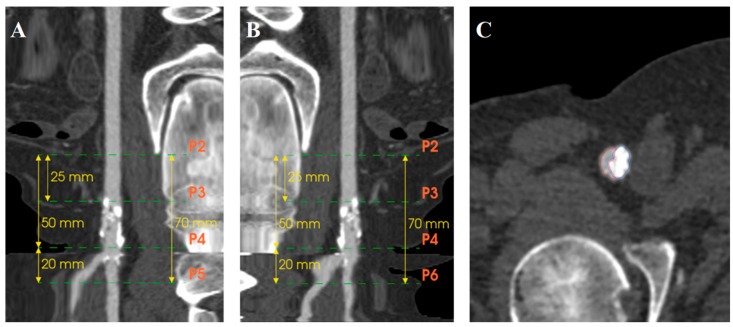
Centerlines and point placements. (**A**) Stretching of the centerline above the DFA. (**B**) Stretching of the centerline above the SFA. (**C**) Centerline analysis of true lumen (red area) and calcifications (blue area).

Computed tomography angiography (CTA) images were retrospectively analyzed using EndoSize software (Therenva, Rennes, France) with a specially developed calcification assessment tool that provided automated measurements through an algorithm differentiating true arterial lumen from calcifications, considering scanner energy parameters and contrast injection density. Briefly, the software creates a region of interest as a tubular structure along extracted centerlines within the arterial lumen. The calcification detection threshold is automatically calculated as the mean Hounsfield Unit (HU) value of voxels surrounding the centerline plus a delta value derived from the standard deviation, establishing a patient-specific threshold that accounts for individual scanner parameters and contrast injection characteristics. This automatically calculated threshold remains adjustable by the operator to optimize calcification detection accuracy. The calcification assessment investigators were blinded to the outcomes. Inter-observer reproducibility was assessed by two independent operators. The intraclass correlation coefficient (ICC) demonstrated good inter-observer agreement for calcification density measurements (ICC = 0.84, 95% CI: 0.71–0.92) and moderate-to-good agreement for volume measurements (ICC = 0.78, 95% CI: 0.62–0.88).The CFA bifurcation was defined as the arterial segment running between the epigastric artery (proximal limit) and two cm after the ostia of the DFA and SFA. Two centerlines were extracted, one from the CFA above the DFA and the other above the SFA. Different points were placed on the centerline (Figure 1). P2 was located at the level of the epigastric artery, P3 midway between the epigastric artery and femoral bifurcation, P4 at the CFA bifurcation, P5 two cm after the ostium of the DFA, and P6 two cm after the ostium of the SFA (Figure 2A,B). Segmentation of the arterial lumen and calcification volume were obtained manually (Figure 2C).

**Figure 3:**
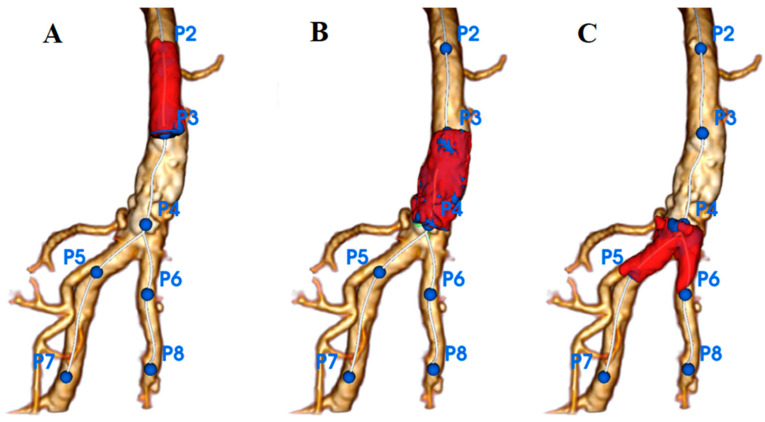
Zones of the CFA and its bifurcation. (**A**) Zone 1: proximal CFA; (**B**) Zone 2: distal CFA; (**C**) Zone 3: femoral bifurcation and ostial segments of the SFA and DFA.

Three zones were defined: Zone 1 proximal CFA (P2–P3), Zone 2 distal CFA (P3–P4), and Zone 3 CFA bifurcation and the ostia segments of the SFA/DFA (P4–P5/P6) (Figure 3). Segmentation of the arterial lumen and calcification volume were obtained manually. For each zone, the minimum, maximum, and median densities (Hounsfield Unit [HU]) and calcification volumes (mm^3^) were measured on preoperative CTA.

### 3.4. Follow-Up Protocol

Follow-up appointments involving a clinical examination, ultrasound scan, and ABI measurement were scheduled one and twelve months after the index procedure. When symptomatic restenosis >50% was detected, CTA was performed.

### 3.5. Endpoints and Definition

The primary endpoint was twelve-month primary patency according to calcification parameters. Primary patency was defined as a binary endpoint based on a duplex ultrasound peak systolic velocity ratio of 2.4 or lower in the absence of clinically driven target lesion revascularization or bypass [19].

Secondary endpoints were one-month primary patency according to the calcification parameters and adjusted twelve-month primary patency (logistic regression) to consider potential cofounding factors, such as the type of stent used and the value of calcifications in the Azema 2 group to mitigate hip mobility.

### 3.6. Statistical Analysis

The analytical unit was the treated limb, as bilateral treatment occurred in some patients during the same procedure. Continuous variables are reported as means ± standard deviation (SD), and categorical variables as counts and percentages. Continuous variables were compared using the Wilcoxon rank-sum test, and categorical variables using chi-square or Fisher’s exact test. The significance threshold was set at α = 0.05. Multiple testing correction was applied using the Benjamini–Hochberg method to control the false discovery rate (FDR). Multivariable analysis used logistic regression. ROC analysis identified optimal calcification thresholds and calculated the area under the curve (AUC). To assess the robustness of the identified threshold, bootstrap validation with 1000 iterations was performed, calculating bias-corrected confidence intervals for the area under the curve and optimal threshold estimates. Statistical analysis was conducted using R software version 4.2.3. Figures were created using Plotly visualization library (V 6.3.0).”

## 4. Results

### 4.1. Patient Characteristics

**Figure 4:**
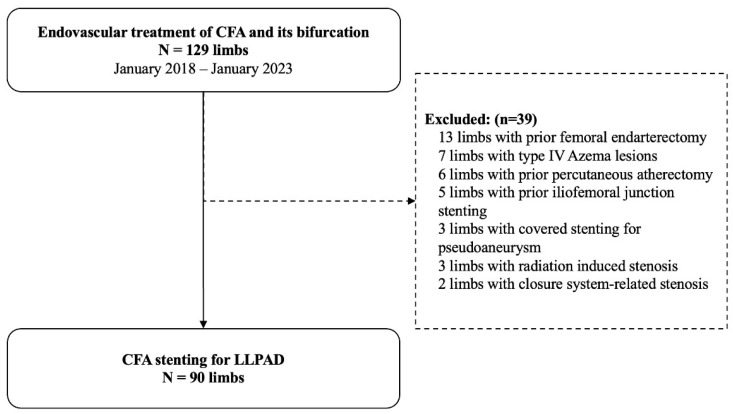
Flowchart. CFA: Common Femoral Artery, LLPAD: Lower Limb Peripheral Artery Disease.

Of the 129 endovascular treatments of the CFA and its bifurcation, 90 limbs (82 patients) required stenting for LLPAD and were included. Patients were excluded if they had prior femoral endarterectomy (n = 13), type IV Azema lesions (n = 7), prior percutaneous atherectomy (n = 6), anterior iliofemoral junction stenting (n = 5), covered stenting for pseudoaneurysm (n = 3), radiation-induced stenosis (n = 3), or closure system-related stenosis (n = 2) (Figure 4). A total of 64 lesions of the CFA and its bifurcation were treated with balloon-expandable stents, and 26 with self-expandable stents. The overall population was 81% (n = 73) male, with a median age of 72 years [66.0; 78.0] (Table 1). The median body mass index (BMI) was 27.3 kg/m^2^ [24.7; 29.5]. A total of 78.9% of CFA lesions were treated for claudication, and 21.1% for CLTI. According to the Azema Classification, 1% (n = 1) of the CFA lesions were type I, 43% (n = 39) were type II, and 56% (n = 50) were type III. Baseline calcification values are presented as medians and interquartile ranges (IQRs) in Table 2.

### 4.2. Primary Outcomes

Twelve-month follow-up data was available for 93.3% of the treated limbs; three patients died and three were lost to follow-up. Primary patency at twelve months was 77.4% (65/84 limbs). A total of 22.6% (n = 19) of limbs had a PSVR > 2.4, and 77.4% (n = 65) had a PSVR < 2.4. Calcification parameters according to PSVR are described in Table 3. Calcification volumes in the three zones were not statistically different between the two groups. The calcification median density (HU) in Zone 2 was significantly higher in patients with PSVR < 2.4, i.e., 1122 (903, 1248) HU compared to 858 (788, 987) HU in patients with PSVR > 2.4 (restenosis), *p* = 0.006, q = 0.021. Other calcification densities in Zones 1 and 3 were not statistically significant. The ratio of calcification volume to arterial lumen volume did not differ significantly between groups.

### 4.3. Secondary Outcomes

**Figure 5:**
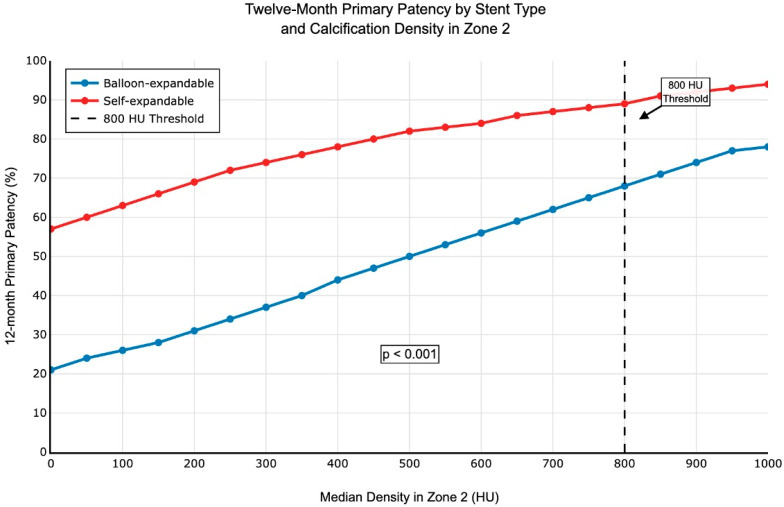
Logistic regression of primary patency according to the calcification parameters in the Azema 2 group. Primary patency rates are shown for balloon-expandable stents (blue) and self-expandable stents (red). Self-expandable stents demonstrate consistently higher patency rates across all calcification density ranges, with the most pronounced benefit observed in heavily calcified lesions (>800 HU). The 800 HU threshold (vertical dashed line) represents the optimal cut-off identified by ROC analysis. HU = Hounsfield Units.

**Figure 6:**
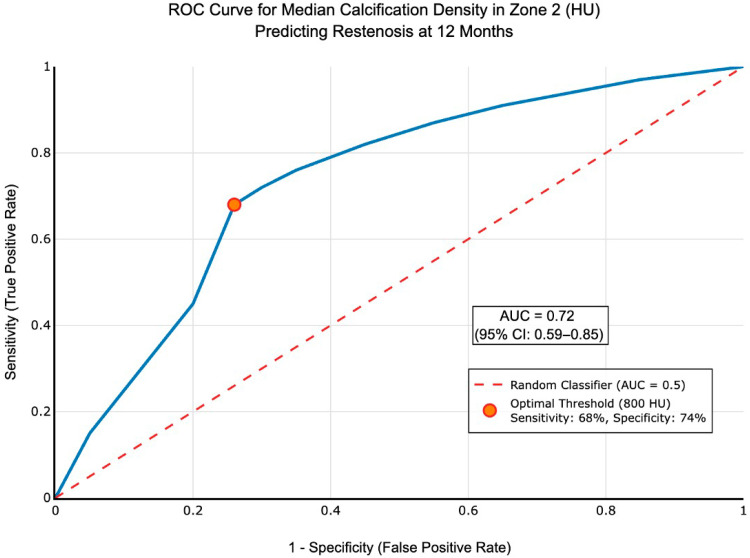
Receiver Operating Characteristic (ROC) curve for median calcification density in Zone 2 (distal CFA) predicting 12-month primary patency. The area under the curve (AUC) is 0.72 (95% CI: 0.59–0.85, *p* < 0.001), indicating good discriminatory ability. The optimal threshold of 800 Hounsfield Units (HU) was identified using the Youden index, providing 68% sensitivity (95% CI: 33.5–79.7) and 74% specificity (95% CI: 63.6–85.5) for predicting treatment failure. The diagonal reference line represents no discriminatory ability (AUC = 0.5). Bootstrap validation with 1000 replications confirmed threshold stability at 821 HU (95% CI: 656–990 HU).

One-month follow-up data were available for 98.9% of limbs (89/90). Nine limbs had a PSVR > 2.4. Calcification values for patency at one month (PSVR < 2.4 or >2.4) are described in Table 4. The maximum, median, and minimum densities in Zone 2 were not significantly different between the two groups at one month. Calcification volume in the three zones was not statistically different between the two groups. Logistic regression analysis including the type of stent used in the Azema 2 group revealed that self-expandable stents had better primary patency than balloon-expandable stents in calcified lesions (*p* < 0.001), Figure 5. Multivariable logistic regression identified calcification density ≥800 HU as an independent protective factor against restenosis (adjusted OR 0.24, 95% CI: 0.08–0.72; *p* = 0.011), corresponding to a 76% reduction in restenosis risk. ROC curve analysis confirmed 800 HU as the density threshold for predicting primary patency (AUC = 0.72, 95% CI: 0.59–0.85, *p* < 0.001), Figure 6. This threshold demonstrated a sensitivity of 68% (95% CI: 33.5–79.7) and specificity of 74% (95% CI: 63.6–85.5) for predicting 12-month treatment failure. The positive predictive value was 42.3% (CI95%), and the negative predictive value was 85.5% (CI95%:). Bootstrap validation with 1000 replications confirmed threshold stability, yielding a threshold of 821 HU (95% CI: 656–990 HU). Patients with calcification densities below 800 HU showed increased risk of restenosis at 12 months.

## 5. Discussion

To our knowledge, this is the first study to quantitatively investigate the relationship between arterial calcification densities and twelve-month primary patency following CFA stenting using automated three-dimensional analysis. Our findings suggest that dense calcifications (≥800 HU) in the distal CFA (Zone 2) may be independently associated with a 76% reduction in restenosis risk, indicating that calcification density rather than volume alone influences clinical outcomes. Our primary finding that calcification density ≥800 HU in Zone 2 protects against restenosis represents a paradigm shift in understanding vascular calcification. This is consistent with recent literature suggesting that the relationship between calcification and endovascular outcomes is more nuanced than previously appreciated. Kaladji et al. [14] demonstrated that severe calcifications were associated with perioperative thrombosis but had a lower rate of restenosis after superficial femoral artery stenting, supporting our observation in the CFA territory. The 800 HU threshold identified in our study may provide a quantitative criterion that warrants further validation. This density threshold likely reflects mature osteoid calcification [17] rather than early inflammatory calcification, as evidenced by the specific involvement of Zone 2—the distal CFA segment proximal to the femoral bifurcation. Traditional assessment of arterial calcification remains difficult, with clinical tools unable to directly monitor underlying arterial remodeling processes [20]. Our zone-specific approach addresses this limitation by providing reproducible, anatomically relevant measurements that correlate with clinical outcomes.

Zone 2 emerged as the critical anatomical segment, which aligns with biomechanical principles. Tijani et al. [21] demonstrated that the CFA is a fixed arterial segment, with Zone 2 showing minimal displacement during hip flexion compared to more proximal and distal segments. This relative immobility during hip movement may explain why calcification density specifically in this region influences patency outcomes, as mechanical stress from hip joint mobility could potentially compromise stent integrity and patency in more mobile segments. The protective effect of dense calcifications likely reflects fundamental changes in the vascular smooth muscle cell (VSMC) phenotype and local microenvironment. Vascular calcification involves VSMC differentiation into osteoblast-like cells, generating matrix vesicles that serve as nidus for calcium–phosphate deposition. However, the density and maturity of these calcifications appear to determine their biological impact. Dense calcifications are associated with a contractile rather than synthetic VSMC phenotype [17,22]. This phenotypic stability may explain the reduced restenosis risk, as synthetic VSMCs are primarily responsible for neointimal hyperplasia following vascular injury [23]. The high-density calcification (≥800 HU) threshold identified in our study likely represents mature osteoid tissue formation, which has been specifically documented in CFA atherosclerotic plaques as distinguishing these lesions from other vascular territories [17,20]. Dense calcifications may create a local microenvironment that inhibits the cellular processes underlying restenosis. Phenotypic switching of VSMCs involves oxidative stress-induced extracellular vesicle release, driving calcification processes [24,25]. Mature calcifications may represent the end-stage organization of this inflammatory cascade, resulting in metabolically quiescent tissue that is resistant to further proliferative responses. The calcium–phosphate crystals in dense calcifications may also physically constrain vessel wall expansion and remodeling, limiting the space available for neointimal proliferation. This mechanical constraint, combined with the reduced cellular activity in densely calcified tissue, could explain the protective effect observed here. Restenosis results from neointimal hyperplasia, which involves VSMC proliferation, migration, and extracellular matrix deposition [26]. Our findings in the CFA territory corroborate observations in the superficial femoral artery. The relationship between calcification and restenosis differs markedly between coronary and peripheral territories. In coronary arteries, calcification is generally associated with increased procedural complexity and worse outcomes [27,28]. The choice of stent type in CFA interventions must account for the unique biomechanical environment of the femoral region. In our study, self-expandable stents showed superior performance compared to balloon-expandable stents in the multivariable analysis. Self-expandable stents offer continued radial force against external compression from hip joint movement and allow for safe arterial access in future procedures [1,2]. Several limitations should be acknowledged. This was a single-center retrospective study with inherent selection biases and a relatively small sample size (ninety limbs). EndoSize software has not been extensively validated against histological standards, and our analysis did not differentiate between medial and intimal calcification. Our analysis did not distinguish between medial and intimal calcification, which may represent different pathophysiological processes. Bootstrap validation with 1000 replications confirmed threshold stability, yielding an optimal threshold of 821 HU (95% CI: 656–990 HU), closely approximating our pre-specified 800 HU cut-off. This validation supports the robustness of our threshold selection and its potential clinical applicability. However, stent selection was based on operator preference and lesion complexity rather than randomized allocation, potentially introducing confounding factors. The continuous radial force and shape memory properties of nitinol may provide biomechanical advantages in the dynamic CFA environment, though randomized trials are needed to confirm these observations. The twelve-month follow-up period may not capture late restenosis events, and time-to-event analysis was not performed. External validation in independent cohorts remains necessary to confirm the generalizability of our findings across different populations and clinical settings. A randomized controlled trial comparing treatment strategies based on calcification density could definitively establish the clinical utility of our 800 HU threshold. Future research should focus on understanding the cellular mechanisms underlying the protective effect and investigating whether similar density-dependent effects exist in other peripheral territories.

## 6. Conclusions

In this study, high density calcifications in Zone 2 (≥800 HU) of the CFA may not compromise the twelve-month primary patency after stenting of the CFA and its bifurcation, and there is a lower rate of restenosis in the most calcified lesions. Multicenter validation and prospective analysis are required for clinical use.

## Figures and Tables

**Table 1:** Baseline characteristics. Values are expressed as medians [IQR]. HU = Hounsfield Units; mm^3^ = cubic millimeters; IQR = interquartile range. Zone 1 = proximal CFA; Zone 2 = distal CFA; Zone 3 = bifurcation and ostial segments of SFA and DFA. Calcification volume/arterial lumen volume ratio expressed as percentage.

Characteristics	N = 90 (%)
Age	72.0 [66.0; 78.0]
Male sex	73 (81)
Hypertension	83 (92)
Dyslipidemia	78 (87)
Diabetes mellitus	43 (48)
Smokers	
Current	19 (21)
Former > 6 months	64 (71)
Never	4 (4)
Coronary artery disease	39 (43)
BMI (kg/m^2^)	27.3 [24.7; 29.5]
eGFR (mL/min/1.73 m^2^)	80.5 [61.2; 92.0]
Statin	80 (89)
Aspirin	62 (69)
Clopidogrel	19 (21)
Ankle brachial index	0.640 [0.490; 0.800]
Rutherford	
1	3 (3)
2	25 (28)
3	43 (48)
4	7 (8)
5	10 (11)
6	2 (2)
Azema Classification	
1	1 (1)
2	39 (43)
3	50 (56)

**Table 2:** Baseline calcification values. Values are expressed as medians [IQR]. HU = Hounsfield Units; mm^3^ = cubic millimeters; IQR = interquartile range. Zone 1 = proximal CFA; Zone 2 = distal CFA; Zone 3 = bifurcation and ostial segments of SFA and DFA. Calcification volume/arterial lumen volume ratio expressed as percentage.

Calcifications	N = 90
Zone 1	
Volume (mm^3^)	186 [75.0; 409]
Minimum density (HU)	584 [442; 711]
Median density (HU)	990 [808; 1148]
Maximum density (HU)	1745 [1390; 2008]
Calcification volume/arterial lumen volume (%)	19.1 [8.14; 28.1]
Zone 2	
Volume (mm^3^)	263 [128; 474]
Minimum density (HU)	640 [529; 773]
Median density (HU)	1025 [884; 1154]
Maximum density (HU)	1896 [1600; 2100]
Calcification volume/arterial lumen volume (%)	20.4 [13.2; 27.6]
Zone 3	
Volume (mm^3^)	76.0 [22.0; 144]
Minimum density (HU)	637 [510; 742]
Median density (HU)	961 [752; 1094]
Maximum density (HU)	1574 [1210; 1920]
Calcification volume/arterial lumen volume (%)	8.86 [3.82; 13.8]

**Table 3:** Calcification values for patency at twelve months. Values are expressed as medians [IQR]. PSVR = peak systolic velocity ratio; mm^3^ = cubic millimeters; HU = Hounsfield Units; IQR = interquartile range. PSVR > 2.4 indicates hemodynamically significant restenosis. *p*-values from Wilcoxon rank-sum test; Q-values represent false discovery rate-adjusted *p*-values using Benjamini–Hochberg method.

Patency at M12	PSVR > 2.4 (N = 19)	PSVR < 2.4 (N = 65)	*p*-Value	Q-Value
Zone 1				
Volume (mm^3^)	121 (75, 238)	234 (72, 428)	0.075	0.20
Minimum density (HU)	561 (431, 712)	588 (452, 708)	0.92	0.92
Median density (HU)	888 (748, 1124)	998 (836, 1142)	0.67	0.72
Maximum density (HU)	1650 (1261, 2092)	1812 (1469, 1982)	0.57	0.66
Calcification volume/arterial lumen volume (%)	12.1 [6.25; 17.1]	19.1 [8.14; 28.1]	0.096	0.91
Zone 2				
Volume (mm^3^)	190 (86, 335)	282 (142, 494)	0.12	0.24
Minimum density (HU)	652 (522, 833)	627 (537, 723)	0.46	0.60
Median density (HU)	858 (788, 987)	1122 (903, 1248)	0.006	0.021
Maximum density (HU)	1911 (1364, 2008)	1907 (1682, 2181)	0.23	0.37
Calcification volume/arterial lumen volume (%)	15.2 [8.7; 21.7]	20.4 [13.2; 27.6]	0.055	0.20
Zone 3				
Volume (mm^3^)	48 (8, 100)	80 (30, 148)	0.058	0.19
Minimum density (HU)	527 (412, 640)	648 (537, 752)	0.041	0.17
Median density (HU)	748 (538, 932)	999 (821, 1132)	0.005	0.061
Maximum density (HU)	1202 (824, 1582)	1631 (1296, 1955)	0.014	0.072
Calcification volume/arterial lumen volume (%)	6.41 [0.915; 11.4]	8.86 [3.82; 13.8]	0.12	0.34

**Table 4:** Calcification values for patency at one month. Values are expressed as medians [IQR]. PSVR = peak systolic velocity ratio; mm^3^ = cubic millimeters; HU = Hounsfield Units; IQR = interquartile range. PSVR > 2.4 indicates hemodynamically significant restenosis. *p*-values from Wilcoxon rank-sum test; Q-values represent false discovery rate-adjusted *p*-values using Benjamini–Hochberg method.

Patency at M1	PSVR > 2.4 (N = 9)	PSVR < 2.4 (N = 80)	*p*-Value	Q-Value
Zone 1				
Volume	227 (149, 293)	185 (72, 416)	0.94	0.99
Minimum density	517 (512, 745)	586 (441, 708)	0.99	0.99
Median density	796 (693, 1078)	994 (825, 1150)	0.49	0.65
Maximum density	1334 (1296, 1776)	1772 (1416, 2017)	0.32	0.55
Calcification volume/arterial lumen volume (%)	12.1 [11.6; 18.5]	15.8 [6.84; 27.0]	0.41	0.63
Zone 2				
Volume	111 (79, 263)	266 (142, 476)	0.18	0.35
Minimum density	595 (463, 759)	644 (537, 778)	0.65	0.76
Median density	859 (709, 926)	1030 (890, 1170)	0.060	0.18
Maximum density	1377 (1351, 1381)	1914 (1698, 2147)	0.014	0.43
Calcification volume/arterial lumen volume (%)	15.5 [7.40; 20.0]	18.3 [11.4; 26.8]	0.33	0.53
Zone 3				
Volume	95 (5, 105)	75 (23, 148)	0.42	0.63
Minimum density	514 (435, 541)	642 (519, 752)	0.11	0.25
Median density	600 (515, 738)	983 (788, 1114)	0.012	0.059
Maximum density	946 (516, 1161)	1596 (1231, 1948)	0.005	0.36
Calcification volume/arterial lumen volume (%)	9.12 [3.24; 13.3]	7.26 [2.48; 13.1]	0.92	1

## Data Availability

The datasets generated and analyzed during the current study are not publicly available due to patient confidentiality and institutional privacy policies but are available from the corresponding author on reasonable request subject to institutional review board approval and establishment of appropriate data use agreements.
